# Profiling Insulin Like Factor 3 (INSL3) Signaling in Human Osteoblasts

**DOI:** 10.1371/journal.pone.0029733

**Published:** 2011-12-28

**Authors:** Alberto Ferlin, Lisa Perilli, Lisa Gianesello, Giuseppe Taglialavoro, Carlo Foresta

**Affiliations:** 1 Section of Clinical Pathology, Department of Histology, Microbiology and Medical Biotechnologies, University of Padova, Padova, Italy; 2 Orthopaedic and Traumatological Clinic, University of Padova, Padova, Italy; Medical College of Georgia, United States of America

## Abstract

**Background:**

Young men with mutations in the gene for the INSL3 receptor (Relaxin family peptide 2, RXFP2) are at risk of reduced bone mass and osteoporosis. Consistent with the human phenotype, bone analyses of *Rxfp2* ^−/−^ mice showed decreased bone volume, alterations of the trabecular bone, reduced mineralizing surface, bone formation, and osteoclast surface. The aim of this study was to elucidate the INSL3/RXFP2 signaling pathways and targets in human osteoblasts.

**Methodology/Principal Findings:**

Alkaline phosphatase (ALP) production, protein phosphorylation, intracellular calcium, gene expression, and mineralization studies have been performed. INSL3 induced a significant increase in ALP production, and Western blot and ELISA analyses of multiple intracellular signaling pathway molecules and their phosphorylation status revealed that the MAPK was the major pathway influenced by INSL3, whereas it does not modify intracellular calcium concentration. Quantitative Real Time PCR and Western blotting showed that INSL3 regulates the expression of different osteoblast markers. Alizarin red-S staining confirmed that INSL3-stimulated osteoblasts are fully differentiated and able to mineralize the extracellular matrix.

**Conclusions/Significance:**

Together with previous findings, this study demonstrates that the INSL3/RXFP2 system is involved in bone metabolism by acting on the MAPK cascade and stimulating transcription of important genes of osteoblast maturation/differentiation and osteoclastogenesis.

## Introduction

Sex hormones have a critical role in bone metabolism [1], [2]. In particular, in the male, androgens are necessary for the gain of bone mass during puberty and its maintenance during adulthood [1], [2]. Another testis-specific, Leydig cell derived hormone with a recently identified role in bone metabolism is Insulin-like factor 3 (INSL3) [3]–[5]. INSL3 is a member of the relaxin-insulin family peptide and it was initially characterized as an essential hormone for the correct descent of the testes into the scrotum during fetal life [3]. Apart from this role in physiological and pathological testicular descent [6], INSL3 has recently gained further attention for its possible roles in adulthood [7], [8]. In fact, INSL3 is produced constitutively but in a differentiation-dependent manner by the Leydig cells under the long-term Leydig cell differentiation effect of LH, and substantial circulating INSL3 levels are present in adult men (500–1000 pg/mL) [3], [9], [10]. Reduced plasma concentrations of INSL3 are seen in situations of undifferentiated or altered Leydig cell status (such as hypogonadism and ageing), and INSL3 has been suggested to be even more sensitive than testosterone to impaired Leydig cell function [3], [8], [9]. INSL3 could be therefore responsible for, or contribute to, some clinical signs of hypogonadism currently attributed to testosterone deficiency.

Following this hypothesis, we recently showed that young men with mutations in the gene for the INSL3 receptor (Relaxin family peptide 2, *RXFP2*) are at risk of reduced bone mass and osteoporosis, despite their testosterone plasma levels are within the normal range [4]. INSL3 induced a significant and dose-dependent increase in cAMP and proliferation of human primary osteoblasts, supporting a RXFP2-mediated role of INSL3 in osteoblasts function [4], [11].

Consistent with the human phenotype, bone histomorphometric and µCT analyses at the lumbar and femoral sites of *Rxfp2* ^−/−^ mice showed decreased bone volume, alterations of the trabecular bone, reduced mineralizing surface, bone formation, and osteoclast surface [4]. These data suggested that the low bone mass phenotype in the *Rxfp2* ^−/−^ mice is linked to functional osteoblast impairment causing little bone formation, little mineralizing surface, and ultimately, a negative balance between bone formation and bone resorption [4].

To better understand the molecular mechanisms by which INSL3 acts on human osteoblasts, in this study we analyzed the effects of this hormone on human osteoblasts on different signaling pathways (intracellular calcium mobilization, protein phosphorylation and gene expression) that could be influenced by a G-protein coupled receptor (GPCR) as RXFP2. This is particularly intriguing in the light of a recent study by Kaftanovskaya *et al*. [12] that demonstrated that β-catenin, a critical protein for normal bone development [13], could be a potential INSL3 target during gubernaculum development, suggesting that INSL3 could exert its action on osteoblasts not only *via* intracellular cAMP regulation.

## Results

### INSL3 stimulates alkaline phosphatase (ALP) production by human osteoblasts

**Figure 1:**
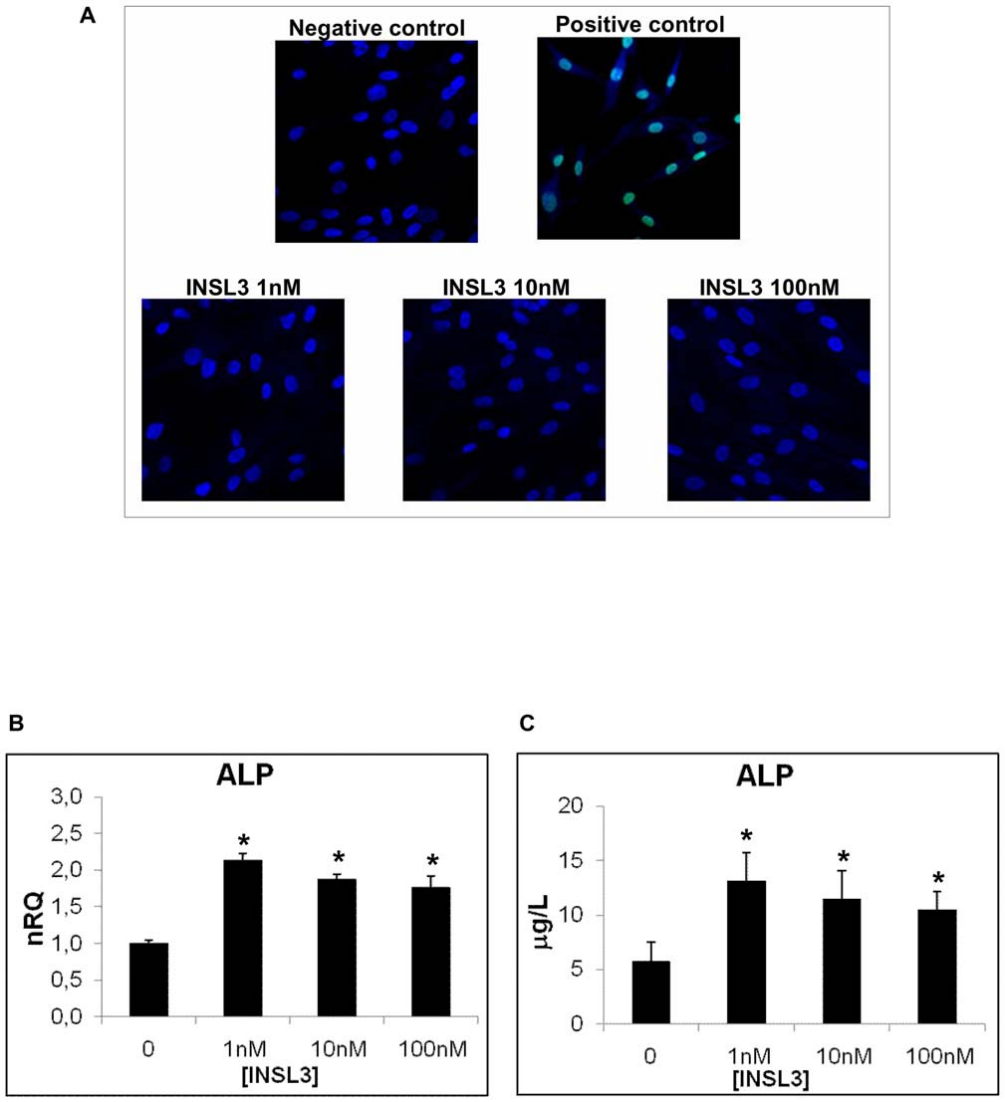
Evaluation of INSL3 toxicity and its effects on ALP production in human osteoblasts. **A**. TUNEL analysis on human osteoblasts stimulated overnight with INSL3 at 1 nM, 10 nM and 100 nM. Positive control is obtained treating cells with DNase. Green fluorescence indicates DNA fragmentation. Nuclei were counterstained with DAPI (Blue). **B**. Quantitative RT-PCR for ALP in human osteoblasts stimulated with INSL3 at different concentrations. Quantification was normalized to the expression of the housekeeping gene β_2_-microglobulin on control (no stimulation). Data are shown as the mean ± standard deviation (SD) of the mean of three different experiments performed in triplicate. **C.** Immunoenzymatic assay of ALP production in human osteoblasts after a 24 hour stimulation with INSL3 at different concentration. Control is obtained without stimulation. Data are shown as the mean ± standard deviation (SD) of the mean of three different experiments performed in triplicate. * P<0.05 vs control.

To test the viability of human osteoblasts stimulated with INSL3 and its possible toxicity we performed a TUNEL test that showed the DNA integrity of osteoblasts and the absence of apoptosis at any INSL3 concentration (1 nM, 10 nM and 100 nM) (Figure 1A).

INSL3 stimulation (at 1 nM, 10 nM and 100 nM) induced a significant increase in the production of ALP, the most important osteoblast marker, as evidenced by quantitative RT-PCR (Figure 1B) and immunoenzymatic assay (Figure 1C).

### INSL3 influences protein phosphorylation but not intracellular calcium concentration

**Figure 2:**
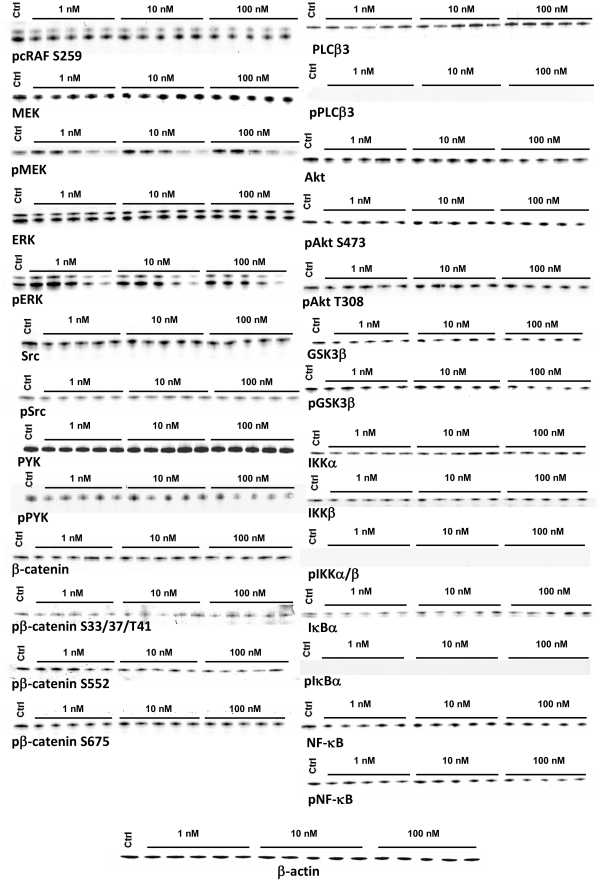
INSL3 effects on protein phosphorylation in human osteoblasts. Western blot analysis for the phosphorylation of proteins involved in MAPK pathway (cRAF, MEK, ERK), integrin pathway (PYK, Src), β-catenin pathway, calcium pathway (PLCβ3), Akt pathway (Akt, GS3β) and NF-κB pathway (NF-κB, IKKα, IKKβ, IκBα). Cells were stimulated with different INSL3 concentrations (1 nM, 10 nM and 100 nM) for a short time period (5, 10, 15, 30 and 45 minutes). β-actin was used as endogenous control to normalize protein quantity. Control is obtained without stimulation. Figures are representative of three independent experiments. Ctrl: Control.

**Figure 3:**
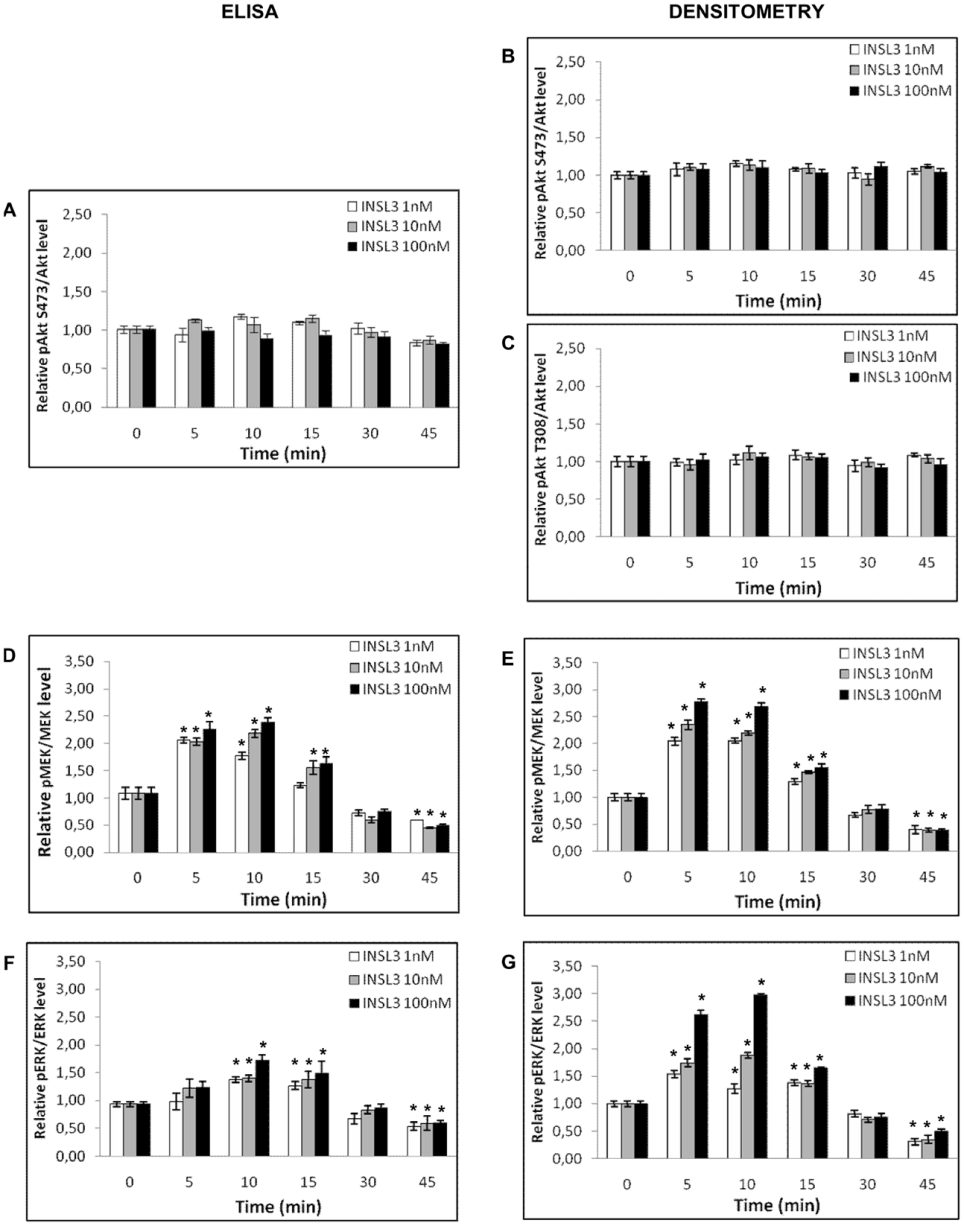
Quantitative phosphorylation analysis of Akt, MEK, and ERK in human osteoblasts induced by INSL3. Quantitative analysis on Akt phosphorylation: **A**. ELISA at S473, **B**. densitometric analysis at S473 and **C**. densitometric analysis at T308. Quantitative analysis on MEK phosphorylation: **D**. ELISA and **E**. densitometric analysis. Quantitative analysis on ERK phosphorylation: **F**. ELISA and **G**. densitometric analysis. ELISA data are shown as the mean ± standard deviation (SD) of the mean of three different experiments performed in triplicate. Densitometric data are shown as the mean ± standard deviation (SD) of the mean of three different experiments. * P<0.05 vs control.

To investigate whether the INSL3/RXFP2 system influences the phosphorylation of several key proteins, we stimulated human osteoblasts with different INSL3 concentrations (1 nM, 10 nM, 100 nM) for short time periods (5, 10, 15, 30, 45 minutes) and then we performed Western blot analysis. MEK and ERK showed a similar trend, with and increase in phosphorylation at 5–15 minutes and then a decrease at 30–45 min in a dose dependent manner (Figure 2). This observation was further supported by ELISA (Figure 3D and F) and densitometric analysis (Figure 3E and G). Integrin key proteins (Src and PYK2), PLCβ3 and β-catenin were not influenced by INSL3 (Figure 2), and similarly there was no change in cRaf inhibitory phosphorylation at S259 [14].

In the same way no variation in phosphorylations were observed for the Akt pathway (Akt and GSK3β) (Figure 2). These data were further supported by ELISA (Figure 3A) and densitometric analysis of Western blotting (Figure 3B and C). Finally, NF-κB signaling (NF-κB, IKKα, IKKβ, IκBα) was not affected by INSL3 (Figure 2). All these results were also confirmed by densitometric analysis (data not shown).

**Figure 4:**
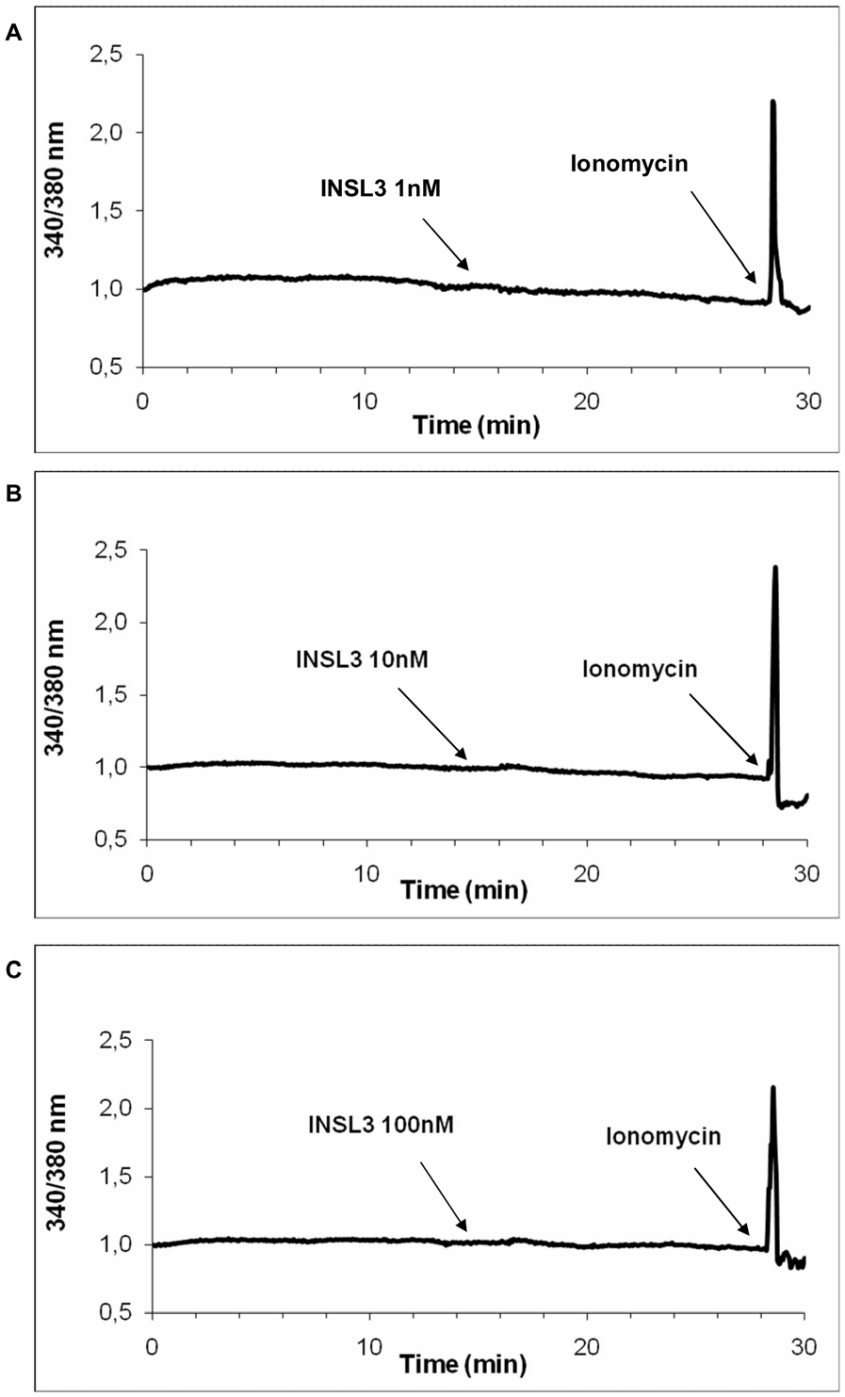
Intracellular calcium evaluation with in human osteoblasts stimulated with INSL3. Calcium mobilization (evaluated by FURA-2) in human osteoblasts after stimulation with **A**. INSL3 1nM, **B**. INSL3 10 nM and **C**. 100 nM. Figures are representative of three independent experiments.

To determine whether INSL3 might act on intracellular calcium concentration, we used the fluorescent calcium indicator FURA-2. No variation in intracellular calcium was observed at any INSL3 concentration (1 nM, 10 nM, 100 nM) (Figure 4). Calcium stability was furthermore confirmed by the absence of PLCβ3 phosphorylation [15] (Figure 2).

### INSL3 modulates gene expression on human osteoblasts and stimulates mineralization

**Figure 5:**
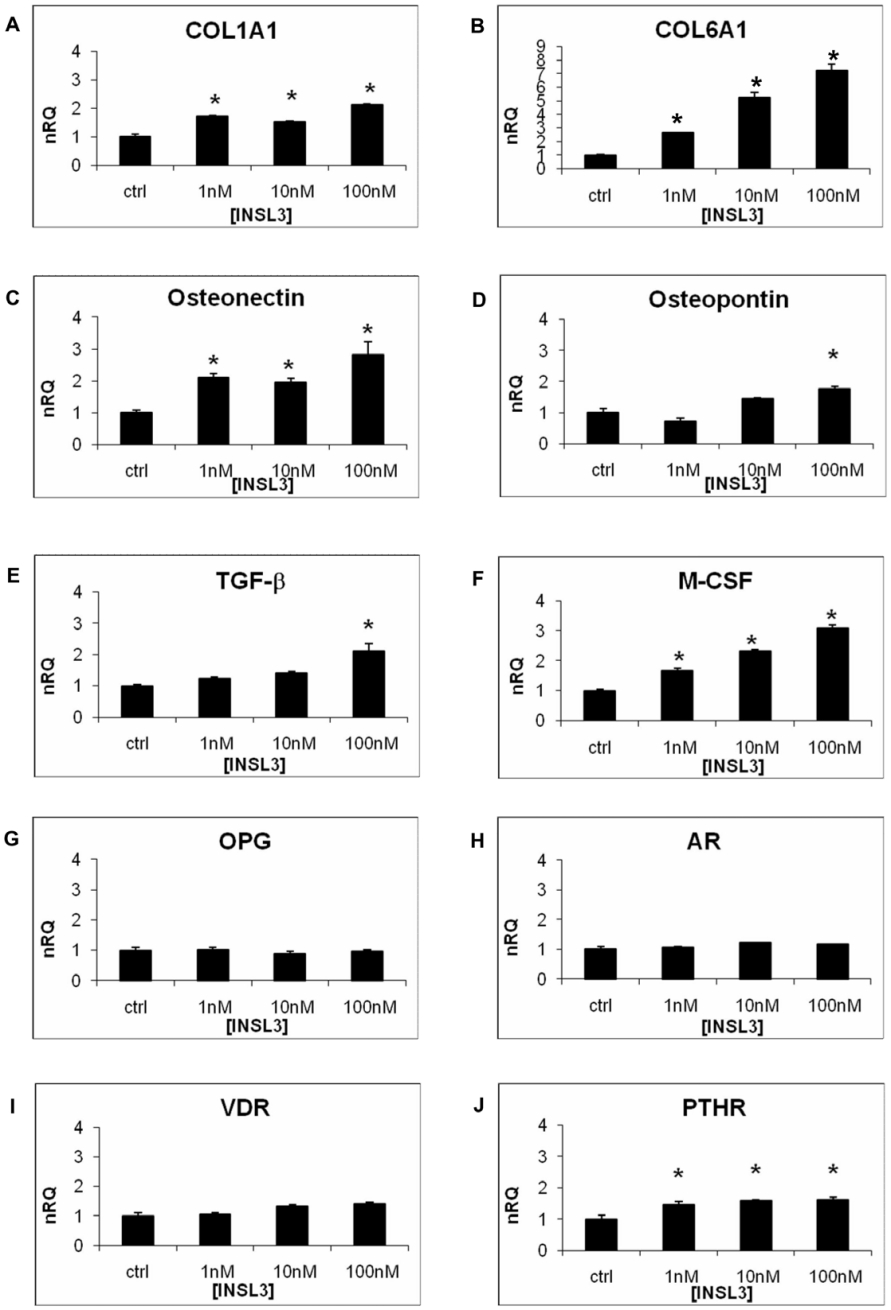
Quantitative RT-PCR on osteoblasts stimulated with INSL3. **A**. *COL1A1*, **B**. *COL6A1*, **C**. *Osteonectin*, **D**. *Osteopontin*, **E**. *TGF-β*, **F**. *M-CSF*, **G**. *OPG*, **H**. *AR*, **I**. *VDR*, **J**. *PTHR*. Quantification was normalized to the expression of the housekeeping gene β_2_-microglobulin on control (no stimulation). Data are shown as the mean ± standard deviation (SD) of the mean of three different experiments performed in triplicate. * P<0.05 vs control. nRQ: normalized Relative Quantity. Ctrl: Control.

Quantitative RT-PCR analysis showed that INSL3 increased the mRNA level of *COL1A1* (*collagen type I, alpha 1*), *COL6A1* (*collagen type VI, alpha 1*) and *osteonectin* (Figure 5A, B and C) at all the concentrations tested. The expression levels of *osteopontin* and *TGF-β* (*transforming growth factor, beta 1*) were increased only at the highest INSL3 concentration (100 nM) (Figure 5D and E). These data showed that INSL3 increases the expression of some osteoblast genes involved in matrix deposition process.

To elucidate if INSL3 could affect bone remodeling, we evaluated its role on the transcriptional levels of cytokines that regulate negatively (*OPG - osteoprotegerin*) or positively (*M-CSF – Macrophage colony stimulating factor* **)** osteoclastogenesis. INSL3 did not modify *OPG* expression (Figure 5G), whereas the effect on *M-CSF* mRNA was clearly dose-dependent (Figure 5F). These results showed that INSL3 positively modulates the osteoclastogenesis process.

Finally, we analyzed the possible effects of INSL3 on gene expression of some important osteoblast receptors. *AR* (*androgen receptor*) and *VDR* [*vitamin D (1,25- dihydroxyvitamin D3) receptor*], expressions were not influenced by INSL3 (Figure 5H and I) whereas *PTHR* (*parathyroid hormone receptor*) mRNA level was increased by INSL3 (Figure 5J). These data showed that INSL3 stimulates only *PHTR* expression, which is involved in the regulation of bone mass.

**Figure 6:**
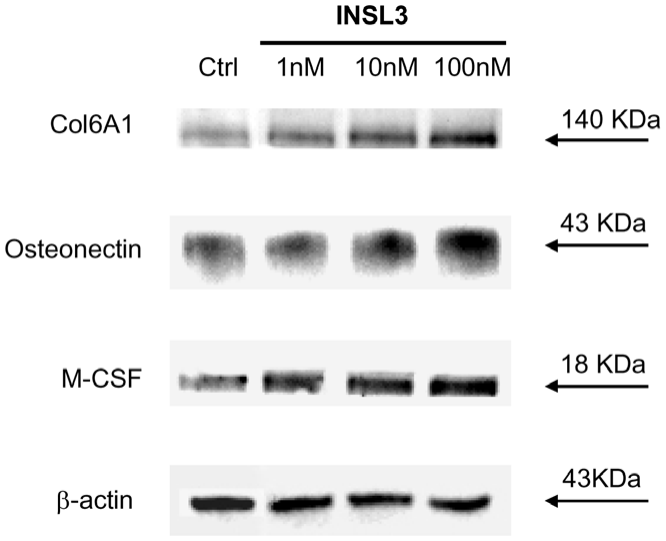
Western blotting for Col6A1, osteonectin, and M-CSF of human osteoblasts stimulated by INSL3. A dose-dependent increase in protein level is evident, confirming quantitative RT-PCR results.

The effect of INSL3 on transcription was confirmed at the protein level by Western blotting for representative genes (Col6A1, osteonectin, and M-CSF) (Figure 6).

**Figure 7:**
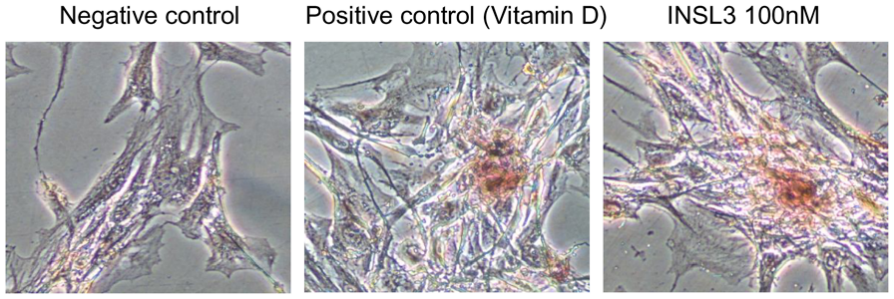
Mineralizing ability of human osteoblasts stimulated with INSL3. Mineralized nodule formation was detected by Alizarin red-S staining and the effect of INSL3 (100 nM) was compared to that of 1,25-vitamin D (100 nM) used as positive control.

Alizarin red-S staining showed bone nodules formation in human osteoblasts treated with INSL3 (Figure 7), confirming the ability of these cells to mineralize the extracellular matrix.

## Discussion

We recently identified the INSL3 receptor RXFP2 on human osteoblasts surface and demonstrated, by human and animal studies, the role of this hormone in the proliferation of osteoblasts and in bone mass regulation [4]. We also showed that INSL3 acts on human osteoblasts through a RXFP2-mediated increase in cAMP [4]. In the light of these data we focused our attention on the molecular events involved in INSL3 signaling in human osteoblasts.

After demonstrating that INSL3 increases the production of the most typical osteoblast protein (ALP), here we demonstrate that the MAPK cascade is the major pathway activated by INSL3 and that this hormone induces the expression of key osteoblast genes involved in osteoblast differentiation, matrix deposition and osteoclastogenesis, and it stimulates mineralization.

**Figure 8:**
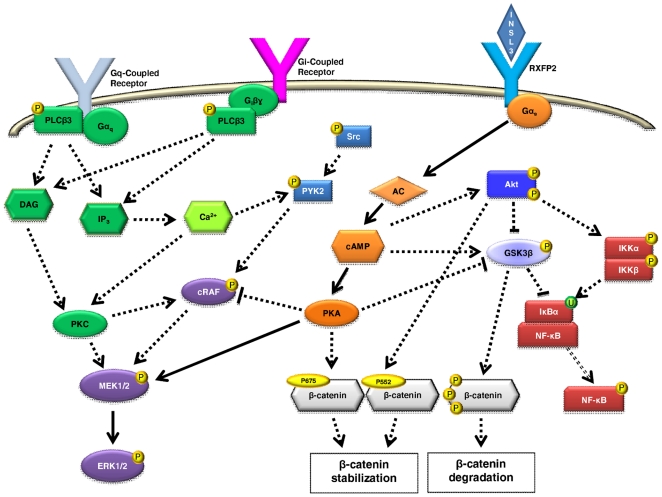
Signaling pathways of INSL3 investigated in human osteoblasts. Continuous lines identify the major pathway stimulated by INSL3 and culminating in MEK/ERK phosphorylation via AC/cAMP/PKA. Dotted lines show excluded pathways in INSL3 signaling from our findings. See text for details. P: phosphorylation, U: Ubiquitination.

GPCRs could activate MEK/ERK and Akt using an intricate signaling network [16]–[18] (Fig. 8), and both these signaling pathways have been implicated in osteoblast differentiation and proliferation [19], [20]. The phosphorylation of ERK is downstream of Raf and MEK (Fig. 8) and this activation allows ERK to regulate transcription of target genes in the nucleus [19]. Cyclic-AMP can regulate MAPK activity via PKA and can either increase or decrease MAPK signaling depending on the different cell type [21] (Fig. 8). Phosphorylation of Akt at T308 and S473 leads Akt to move to cytoplasm and nucleus, where it phosphorylates and activates target proteins involved in different cellular functions [22] (Fig. 8). Cyclic AMP can also regulate Akt phosphorylation and activation [21] (Fig. 8). The kinase GSK3β is downstream of Akt [23] and is constitutively active [17]. GSK-3β can be inactivated through the phosphorylation at S9 by Akt [17] or by cAMP [24] (Fig. 8). Moreover, Akt could act, at least in part, through IKKα to activate canonical NF-κB activity [25] and GSK3β could suppress NF-κB activity by inhibiting IKK and stabilizing IκB [26] (Fig 8). The analysis of these different intracellular signaling pathway moleculaes and their phosphorylation status demonstrated that MEK/ERK phosphorylation is the major pathway involved in INSL3 signaling in human osteoblasts. Considering also our previous results [4], we can assume that the MAPK pathway is stimulated by AC/cAMP/PKA.

However, we considered also other pathways that could activate MAPK signaling, such as cRaf inhibition at S259 [14], PYK2/Src [27], [28], and calcium (Fig. 8). Our data showed that INSL3 does not affect these signaling molecules, nor it induces calcium as second messenger *via* PLCβ3 phosphorylation (G protein- or βγ- mediated) or by direct Ca^2+^/PKC induction (Fig. 8) [15]. Taken together, these data demonstrate that MEK/ERK phosphorylation is the main pathway activated in human osteoblasts by INSL3. This finding fits well with the known effect of MAPK signaling in osteoblast proliferation/differentiation [20], and further supports our previous results [4].

We also examined the Wnt/β-catenin signaling pathway, one of the most extensively studied pathways with direct relevance to basic bone biology [29]. The importance of this pathway in bone formation is undeniable, and it has been demonstrated that the activation of the β-catenin signaling leads to increase bone mass, while suppression results in bone loss [29]. β-catenin could be phosphorylated by GSK3β at S33/S37/T41 or by other kinases as PKA at S675 and Akt at S552 [30] (Fig. 8). Furthermore, it has been recently reported a possible link between INSL3 and β-catenin during gubernaculum development [12]. However, we excluded this pathway, as our data indicated that INSL3 does not influence β-catenin phosphorylation in human osteoblasts. The different results obtained by Kaftanovskaya in gubernacular cells [12] could be due to a crosstalk of pathways that are not involved in bone metabolism or to differences between the two different species (mouse and human).

We next evaluated whether INSL3 stimulates genes involved in osteoblast proliferation/differentiation, matrix deposition and osteoclastogenesis. Other than an important effect on ALP production, we observed a significant effect of INSL3 on the expression of genes involved in the mineralization process, such as COL1A1, COL6A1, osteonectin, osteopontin, and TGF-β. The interaction among osteogenic-related molecules such as COL1A1 is well known as being related to matrix mineralization [31] and COL6A1 has been suggested to play an important role in matrix-matrix interaction and in the construction of the extracellular structure. In some cases of osteoporosis, type VI collagen significantly decreases in bone suggesting that type VI collagen may be important for osteoid structure of bone [32]. Osteonectin is the most abundant non-collagenous protein of developing bone and its high levels in forming bone may reflect a high proliferative potential of the functional osteoblast [33], [34]. Osteopontin comprises about the 2% of the non-collagenous protein in bone [35] and it has important roles in bone turnover serving as attachment for osteoclasts activating the resorption cascade [36]. TGF-β is produced by osteoblasts and regulates the proliferation and differentiation of osteoblasts both *in vitro* and *in vivo* [37] by regulating the production of different genes such as those of the bone specific extracellular matrix proteins including type I collagen [38]. INSL3 could therefore have an important role in matrix deposition as it stimulates the expression of genes coding for collagenous and non–collagenous proteins. These data well agree with the finding that INSL3-stimualted osteoblasts are fully differentiated and are able to mineralize the extracellular matrix. Furthermore, the increased expression of osteonectin and osteopontin underlines the effect of this hormone in the cellular proliferation process.

We previously reported also a reduced osteoclasts population in *Rxfp2* ^−/−^ mice [4], so here we analyzed the effects of INSL3 on osteoblasts/osteoclasts crosstalk. OPG indirectly inhibits osteoclast proliferation and activity by blocking the interaction of RANKL (produced by the osteoblast) with its membrane-bound osteoclast receptor RANK [39], while M-CSF stimulates osteoclastogenesis by binding with the c-FMS receptor on osteoclast surface [13]. We found that INSL3 seems to modulate the osteoclast activity by stimulating osteoclastogenesis mainly acting on M-CSF expression rather than OPG. Although we did not analyze RANKL expression, which is needed to complete osteoclast differentiation, these data suggest a possible mechanism of action by which INSL3 indirectly affects maturation/differentiation of osteoclasts, and well agree with our previous findings on *Rxfp2* ^−/−^ mice [4].

Finally, we evaluated whether INSL3 could influence the expression of genes coding for important osteoblast receptors, such as the androgen receptor (AR) [40], vitamin D receptor (VDR) [41], and parathyroid hormone receptor (PTHR). Only the latter is positively regulated by INSL3 and these data again well agree with our previous experiments on *Rxfp2* ^−/−^ mice [4], as PTH stimulates osteoclast-mediated bone resorption indirectly *via* the activation of the PTHR on osteoblasts [42].

Taken together, the findings of the present study shed light into the molecular and cellular events regulated by INSL3 in human osteoblasts and into the bone phenotype of men and mice with disrupted INSL3 signaling previously described by our group [4]. In fact, young adult men with inactivating mutations of the INSL3 receptor have reduced bone mass and higher risk of osteopenia and osteoporosis despite normal testosterone and INSL3 plasma concentrations [4]. Rxfp2-deficient mice have decreased bone volume and alterations of the trabecular bone as a consequence of a functional impairment of the osteoblasts leading to reduced mineralizing surface, bone formation rate and osteoclast surface [4].

The identification of osteoblasts as INSL3 responsive cells and the association between mutations in the INSL3 receptor gene and reduced bone density open new perspectives in male osteoporosis. Our results demonstrate for the first time that the INSL3/RXFP2 system exerts its action on human osteoblasts not only *via* cAMP but also activating MAPK pathway. Furthermore, the present study confirmed that this hormonal signaling plays a dual role in bone metabolism, inducing both osteoblasts maturation and osteoclasts differentiation.

## Materials and Methods

### Cell culture

The primary explant technique from femoral heads discarded during three arthroplasty (performed by GT) was used to obtain human osteoblast cultures as previously reported by Velasco et al. [43]. Their use for in vitro scientific research does not require ethics approval from the Institutional Review Board. In brief, bone fragments were seeded into T-25 plastic flasks containing DMEM-F/12 (Euroclone) supplemented with 10% FBS (Euroclone), 50 µg/ml ascorbic acid (Sigma-Aldrich), 10^−8^ M dexamethasone (Sigma-Aldrich), and 10 mM β-glycerolphosphate (Sigma-Aldrich). This allowed osteoblastic precursor cells to migrate from the fragments, differentiate and proliferate. After confluence, cells were trypsinized and cultured in the same medium. Third passage cells were used for the evaluation of the osteoblastic phenotype with alkaline phosphatase (ALP) staining and for experiments. Cytochemical ALP staining was performed directly on cell culture plates using a commercial kit (Sigma-Aldrich) to confirm their osteoblast phenotype. Cells were observed with a light microscope and the presence of ALP was indicated by red precipitates. Cells with almost the 80% of ALP positivity were stimulated with human INSL3 (Phoenix Pharmaceuticals, Burlingame, CA, USA) (1 nM, 10 nM and 100 nM) overnight for TUNEL assay, ALP activity, Western blot, and quantitative real-time PCR, or for a shorter period (from 5 to 45 minutes) for Western blot and ELISA analysis. For the Alizarin Red Staining (ARS) cells were incubated for 15 days with INSL3 (1nM, 10nM, 100nM) or Vitamin D (100nM) changing the medium every 3–4 days. The employed INSL3 concentrations bracketed the dissociation constant of INSL3 for its receptor RXFP2 [44].

### Analysis of apoptosis: TUNEL Assay

Cells were fixed for 25 min in 4% paraformaldehyde at room temperature. Apoptosis (DNA integrity) was evaluated at the single-cell level using the DeadEnd™ Fluorometric TUNEL System (Promega, Milan, Italy) according to the manufacturer's instructions. Positive control was represented by pre-treatment with DNase following manufacturer's instructions. Nuclei were counterstained using DAPI (Boehringer). Fluorescently labeled fragments were seen under a fluorescent microscope (Leica TCP SP5). All images were acquired using an oil-immersion 63X objective.

### Bone specific alkaline phosphatase (ALP) determination

Cell lysates were obtained incubating cells with a detergent-containing buffer (0.5% Triton X-100 (Sigma-Aldrich) in 0.1 M Tris-HCl (Carlo Erba) pH 7.8 for 15 min at 37°C.

To determine intracellular ALP concentration we used Ostase BAP Immunoenzymetric Assay (Pantec) following manufacture instructions. Data are shown as the mean ± standard deviation (SD) of the mean of three different experiments performed in triplicate.

### Western blot analysis

After treatment with the hormone, cell lysates were obtained by a physical procedure (freezing in liquid nitrogen and defrosting in water at 37°C) into lysis buffer containing protease inhibitor. Lysates were denatured with SDS and 2-β-mercaptoethanol, boiled and then fractionated using SDS-PAGE gel (Bio-Rad). After blotting onto Hybond ECL Nitrocellulose Membrane (Perkin Elmer) and blocking with a 5% milk solution (Bio-Rad), blots were incubated at 4°C with the primary antibody overnight. The following rabbit primary antibodies from Cell Signaling Technology, were used: anti-Src (1∶500; 2123), antiphospho-Src (1∶500; 2105), anti-Akt (1∶500; 4691), antiphospho-Akt Ser473 XP (1∶500; 4060), antiphospho-Akt Thr308 (1∶250; 2965), anti-ERK1/2 (1∶500; 4695), antiphospho-ERK1/2 Thr202/Tyr204 (1∶500; 4370), anti-MEK1/2, (1∶250; 9122), antiphospho-MEK 1/2 Ser217/221 (1∶250; 9154), anti-GSK3β (1∶500; 9315), antiphospho-GSK3β (1∶500; 9323), antiphospho-cRAF S259 (1∶500; 9421), anti-NF-κB (1∶500; 4764), antiphospho-NFκB (1∶500; 3033), anti-IKKα (1∶500; 2682), antiphospho-IκBα (1∶500; 2859), anti-IKKβ (1∶500; 2678), antiphospho-IKKα/β (1∶500; 2697), anti-PYK (1∶500; 3292), antiphospho-PYK (1∶100; 3291), anti-β-catenin (1∶500; 9582), antiphospho-β-catenin S552 (1∶500; 9566), antiphospho-β-catenin S675 (1∶500; 9567), antiphospho-β-catenin S33/37/T41 (1∶200; 9561). The mouse antibody anti-IκBα (1∶500; 4814) from Cell Signaling Technology was also used. The following rabbit primary antibodies from Santa Cruz Biotechnology, were used: anti-PLCβ3 (1∶100; sc-13958), antiphospho-PLCβ3 (1∶50; sc-34392), anti-MCSF (1∶100; sc-13103), anti-osteonectin (1∶100, sc-25574), anti-Col6A1 (1∶100, sc-20649). Mouse antibody vs β-actin (1∶500; sc-47778 Santa Cruz Biotechnology) served as an internal control. Antibody binding to the membrane was detected using a secondary antibody (goat anti-rabbit IgG 1∶5000; Perkin Elmer or goat anti-mouse IgG 1∶5000 Calbiochem) conjugated to horseradish peroxidase and visualized using enzyme-linked chemiluminescence (ECL, Perkin Elmer) with the Chemidoc XRS System (Bio-Rad). Densitometric analysis was performed with the Quantity One 4.6.9 software (Bio-Rad). Data are shown as the mean ± standard deviation (SD) of the mean of three different experiments performed in triplicate.

### ELISA

Protein extraction for the ELISA assay was performed with the suggested Cell Lysis Buffer (Cell Signaling Technology). The quantitative evaluation of phosphorylations were performed with commercial ELISA form Cell Signal Technology (Akt1 - BK7170, pAkt1 S473 - BK7160, MEK1 - BK7165, pMEK1 - BK7175, ERK - BK7177, and pERK - BK7050) following manufacturer's instructions. Data are shown as the mean ± standard deviation (SD) of the mean of three different experiments performed in triplicate.

### Intracellular calcium concentration measurement

For intracellular calcium measurement, osteoblasts were grown on round glass coverslips and then loaded with 5 µM FURA-2 (Invitrogen) for 30 minutes at 37°C in saline medium added with 2 µl Pluronic Acid (Invitrogen) and 250 µM Sulfinpyrazone (Sigma-Aldrich). Fluorescence was monitored with an Olympus IX 81 inverted microscope by recording and emission signal at 510 nm at 3 seconds intervals excitating at 340 nm and 380 nm. Cellular vitality was evaluated adding 200 nM Ionomycin (Invitrogen). Experiments were performed three times in triplicate.

### RNA isolation and cDNA synthesis

Total RNA was isolated with RNA-Bee reagent. The amount of RNA isolated was determined by spectrometry at 260 nm. Total RNA was used for first-strand cDNA synthesis using the Superscript III RT kit (Invitrogen) according to the manufacturer's instructions. cDNAs were tested by PCR using specific oligonucleotide primers for the housekeeping gene *β-Actin* (*β-Actin* forward 5′-CACTCTTCCAGCCTTCCTTCC-3′ *β-Actin* reverse 5′-CGGACTCGTCATACTCCTGCTT-3′).

### Quantitative RT-PCR

Quantitative expression of typical osteoblast genes was evaluated with TaqMan Gene Expression Assays (Applied Biosystems): *Alkaline phosphatase* (*ALP* – Hs00758192_m1), *Vitamin D Receptor* (*VDR* - Hs01045840_m1), *Collagen type I* (*COL1A1* - Hs00164004_m1), *Collagen type VI* (*COL6A1* - Hs00242448_m1), *Osteoprotegerin* (*OPG* - Hs00171068_m1), *Osteonectin* (*ON* - Hs00234160_m1), *Osteopontin* (*OP* - Hs00167093_m1), *TGF-β* (Hs00852894_g1), *Macrophage Colony Stimulatin Factor* (*M-CSF* - Hs00174164_m1), *Androgen Receptor* (*AR* - Hs00171172_m1) and *Parathyroid Hormone Receptor* (*PTHR* - Hs00174895_m1). Thermal cycling included initial steps at 50°C for 2 min and 95°C for 10 min, followed by 40 cycles at 95°C for 15 sec and 60°C for 1 min. The fluorescence of the double-stranded products was monitored in real time. The cDNA was amplified and quantified using a Sequence Detection System SDS 7900HT (Applied Biosystems). Data were normalized to an internal housekeeping gene, using the TaqMan Human β_2_-microglobulin assay (Hs00187842_m1), and then on control osteoblasts without INSL3 stimulation. Experiments were performed three times in triplicate. Data elaboration was performed as relative quantification analysis using the ΔΔCt method. Data are shown as the mean ± standard deviation (SD) of the mean of three different experiments performed in triplicate.

### Alizarin Red-S Staining

Osteoblasts cultured in 6-well plates were washed with PBS and fixed in 4% (v/v) formaldehyde (Sigma–Aldrich) at room temperature for 20 min. Cells were then washed twice with excess deionized water prior to addition of 2% alizarin red-S (Sigma–Aldrich) (pH 4.1) per well. Plates were incubated at room temperature for 30 min with gentle shaking. After aspiration of the unincorporated dye, wells were washed four times with deionized water while shaking for 5 min. Brown/red staining was visualized using an inverted microscope (Nikon), and the representative pictures were photographed. Experiments were performed in triplicate.

### Statistical analysis

Differences in data obtained in real-time gene expression experiments, proliferation assay, densitometric analysis, ELISA assay and ALP activity test between controls and osteoblasts stimulated with INSL3 at different concentration were determined by paired two-tailed Student's *t*-test after acceptance of normal distribution of the data with the Kolmogorov–Smirnov test. P<0.05 was considered as statistically significant.
